# Deep learning‐based multimodal fusion network for segmentation and classification of breast cancers using B‐mode and elastography ultrasound images

**DOI:** 10.1002/btm2.10480

**Published:** 2022-12-28

**Authors:** Sampa Misra, Chiho Yoon, Kwang‐Ju Kim, Ravi Managuli, Richard G. Barr, Jongduk Baek, Chulhong Kim

**Affiliations:** ^1^ Department of Electrical Engineering, Convergence IT Engineering, Mechanical Engineering, Medical Device Innovation Center, and Graduate School of Artificial Intelligence Pohang University of Science and Technology Pohang South Korea; ^2^ Daegu‐Gyeongbuk Research Center Electronics and Telecommunications Research Institute (ETRI) Daegu South Korea; ^3^ Department of Bioengineering University of Washington Seattle Washington USA; ^4^ Department of Radiology Northeastern Ohio Medical University Youngstown Ohio USA; ^5^ School of Integrated Technology Yonsei University Seoul South Korea

**Keywords:** breast ultrasound images, ensemble learning, image classification, image segmentation, strain ultrasound elastography, transfer learning

## Abstract

Ultrasonography is one of the key medical imaging modalities for evaluating breast lesions. For differentiating benign from malignant lesions, computer‐aided diagnosis (CAD) systems have greatly assisted radiologists by automatically segmenting and identifying features of lesions. Here, we present deep learning (DL)‐based methods to segment the lesions and then classify benign from malignant, utilizing both B‐mode and strain elastography (SE‐mode) images. We propose a weighted multimodal U‐Net (W‐MM‐U‐Net) model for segmenting lesions where optimum weight is assigned on different imaging modalities using a weighted‐skip connection method to emphasize its importance. We design a multimodal fusion framework (MFF) on cropped B‐mode and SE‐mode ultrasound (US) lesion images to classify benign and malignant lesions. The MFF consists of an integrated feature network (IFN) and a decision network (DN). Unlike other recent fusion methods, the proposed MFF method can simultaneously learn complementary information from convolutional neural networks (CNNs) trained using B‐mode and SE‐mode US images. The features from the CNNs are ensembled using the multimodal EmbraceNet model and DN classifies the images using those features. The experimental results (sensitivity of 100 ± 0.00% and specificity of 94.28 ± 7.00%) on the real‐world clinical data showed that the proposed method outperforms the existing single‐ and multimodal methods. The proposed method predicts seven benign patients as benign three times out of five trials and six malignant patients as malignant five out of five trials. The proposed method would potentially enhance the classification accuracy of radiologists for breast cancer detection in US images.

## 1 INTRODUCTION

Breast cancer is the most common and second leading cause of cancer death among women. Ultrasound (US) is one of the key imaging modalities to diagnose breast lesions.^1^ US imaging is used in automated breast lesion classification, segmentation, and detection tasks over the previous decades, thus facilitating patient care. The US imaging is safe, cost‐effective, convenient, and sensitive to breast tumors located in dense areas.^2^ To further improve the US diagnosis accuracy, researchers have investigated functional features in breast tissues using US elastography (e.g., shear‐wave elastography [SWE‐mode] and strain elastography [SE‐mode]). In general, benign breast lesions have a tendency to be stiffer than normal breast tissue. The SE‐mode US images can indicate the stiffness of the tissue for differentiating benign from possible malignant lesions.^3^ The B‐mode US images primarily indicate fundamental information, the US elastography images can give complementary functional knowledge. Clinically, the US modality tries to achieve high specificity while maintaining the best sensitivity using features from both B‐mode and elastography images. High specificity reduces the number of unnecessary biopsies, subsequently improving the quality of the healthcare provided to the patients.

Many promising computer‐aided diagnosis (CAD) techniques have been developed to facilitate clinicians in identifying benign from malignant lesions. However, most of them suffer from the following limitations:

1. Most researchers developed segmentation and classification models based on only B‐mode US images. Few multimodal classification methods have been developed utilizing B‐mode and Doppler^4^ or B‐mode and SE‐mode.^5^, ^6^ However, the utilization of B‐mode and SE‐mode US images for segmentation is yet to be explored.
2. Most existing deep learning (DL) based integration methods^5^ first train models and then combine results with ensemble learning. In other words, the existing method was not able to train each deep learning model and the ensemble model at the same time.^5^ As per our knowledge, there is no DL‐based model reported to date which can simultaneously learn complementary information from B‐mode and SE‐mode US images.
3. Unfortunately, existing multimodal deep learning methods^6^ do not pre‐determine the importance of each input modal and thus give each modal the same weight. Each modal has a different contribution to the output, which is not currently considered. We present an ideal model that gives more weight to essential modals to increase diagnostic accuracy.

## 2 RELATED WORK

### 2.1 Single‐modality work

The CAD uses a computerized program to help the radiologist by providing a second opinion with image analysis and diagnosis. Most of the current CAD techniques for clinical use mainly utilize B‐mode images.

Several automatic segmentation methods have been developed to segment lesions. Early CAD methods used traditional machine learning (ML) algorithms. These algorithms included thresholding, edge detection, region‐based segmentation, and clustering techniques.^7^

Recently, DL model‐based image segmentation models have developed with excellent performance. U‐Net is one of the very popular CNNs architectures for medical image segmentation.^8^ Variations of U‐Net models have been developed recently for medical image segmentation methods due to the complexity and diversity of medical images.^9^

In addition to the segmentation of lesions, CAD methods have also been used to differentiate malignant from benign masses in ultrasound breast images to improve diagnostic accuracy. A support vector machine (SVM) based classifier was designed in Ref. 10 to distinguish whether the tumor was benign or malignant. Zheng et al.^11^ used neural networks and *k*‐means unsupervised classification to identify the tumors. A Fisher linear discriminant analysis and mutual information are utilized in Ref. 12 for classifying the obtained ranked features. However, experimental results prove that these methods are inappropriate for clinical application because they need handcrafted features.

The DL‐based CNNs have also been applied successfully to solve several medical image classification problems. The extracted features from CNN represent complex hierarchical interpretations of inputs.^13^ One of the limitations of training CNN from scratch is it needs a huge annotated image data set, which is unfortunately not available in the medical imaging domain. So, using small data to train CNN leads to an overfitted model. Transfer learning (TL)^14^ and data augmentation often address the limited data problems. Han et al. utilize the GoogLeNet model to differentiate the distinctive types of lesions and nodules from the US imaging. TL‐based CNN models are developed in Refs. 15, 16 to classify lesions in US breast images. A block‐based method and a pre‐trained VGG‐19 model are used in Ref. 17 to segment the lesion and classify the benign tumor from malignant, respectively. However, existing methods discussed so far used B‐mode images only and did not incorporate, multimodal information, potentially enhancing the diagnosis.

### 2.2 Multi‐modality work

Multimodal US imaging, where information from different modes is combined for better diagnosis, has been steadily rising in recent decades owing to the technological advances in US machines. Few researchers developed ML‐based fusion methods to handle multimodal information for segmentation and classification.

Pons et al.^18^ developed a lesion segmentation framework that considers intensity (B‐mode) and strain information. They used a maximum a posteriori approach and Markov random field.

The most common strategy using multi‐modality data is to obtain features from several modalities individually and then combine features for classification. For example, a logistic regression classifier was applied in the manually cropped grayscale and Doppler US images to classify breast lesions.^4^ Zhang et al.^19^ extracted morphological and texture features from segmented B‐mode and SWE‐mode US images. They then applied a deep polynomial network to identify malignant from benign breast tumors. Gong et al.^5^ combined multi‐view deep mapping features with SVM classification using B‐mode and SE‐mode images. These methods use separate networks to extract multimodal information. So, the number of parameters will be huge, and even overfitting can happen.

Gu et al.^20^ designed a DL model comprising multi‐fusion layers to obtain modal‐specific features and correlate information, respectively. Huang et al.^21^ developed a framework to utilize B‐mode, SWE‐mode, Doppler‐mode, and SE‐mode breast US images to assist breast cancer diagnosis. However, these methods require training the model for deep features and classifiers separately to achieve ensembled classification results. In addition, they are also limited to a specific number of modalities.

A strategy for classifying benign and malignant tumors utilizing ensemble TL and a combination of B‐mode and SE breast US images was also previously presented in Ref. 6. Since no segmentation method was employed to automatically segment the cancer lesion, the lesion was identified and cropped manually based on the radiologist's guidance. The manually cropped B‐mode and SE‐mode breast US images were then integrated stack‐wise to extract features for diagnosis. Manually segmenting lesion is tedious and very time‐consuming given the size of data set needed for deep learning study. Thus, automatic segmentation of cancerous region would be a significant enhancement for the deep learning study.

In this study, we propose a weighted multimodal U‐Net (W‐MM‐U‐Net) model for automatically segmenting the lesions using both B‐mode and SE‐mode US images. We then study the benefit of segmentation for improved classification of benign and malignant breast lesions. A baseline classification model is developed without using any segmentation for comparing the performance. We also compare manual (by the radiologist) and automatic (by the proposed method) segmentation to determine classification performances. We use the weighted‐skip connection method to emphasize the importance of different modalities. A multimodal fusion framework (MFF) is also proposed to classify benign from malignant lesions. The MFF consists of VGG‐16 and EmbraceNet models.^22^ Pre‐trained VGG‐16 models are fine‐tuned using B‐mode and SE‐mode images and the results are fused using EmbraceNet model. The features of each modality to a representation appropriate for fusion, and the latter merges the representation of various modalities in a probabilistic way.

The key contributions of work presented here are as follows:

*Segmentation*:

1. B‐mode and SE‐mode US images are employed in the proposed DL‐based segmentation method. A color input processing layer is also added to the network to use the stiffness information present in color.
2. The contraction path uses a 2D multimodal U‐Net (MM‐U‐Net) backbone.^8^ A dense connection technique is used at the contraction path of the 2D MM‐U‐Net, which densely connects information from multiple modalities.^23^
3. Extended paths in the network are connected using a weighted skip connection. The skip connections recover lost spatial information and weight‐relevant modal inputs in the contraction process. We use the classification network to obtain the weight vector value showing the importance of each modal before utilizing it in the segmentation network.

*Classification*:

1. We introduce a trainable end‐to‐end MFF to ensemble deep features of the DL‐model for breast US lesion classification.
2. The features from the DL models fine‐tuned using B‐mode and SE‐mode US images are ensembled using the EmbraceNet model. Unlike other ensembled methods, the proposed EmbraceNet‐based ensembled model reflects cross‐modal correlations and efficiently prevents overfitting because of its regularized learning process. During training, the embracement process was operated probabilistically to select partial info from each modality for integration.
3. Here, we utilized images belonging to the B‐mode and SE‐mode. The modal is flexible to incorporate any number of modalities. The experimental results demonstrate that the proposed EmbraceNet‐based MFF model outperforms the state‐of‐the‐art single‐model and multimodal methods.

The multimodal segmentation network (MM‐U‐Net) achieved a better dice score (0.77) than the single‐modal segmentation network (0.72 using only B‐mode and 0.76 using only SE‐mode). The proposed W‐MM‐U‐Net achieved the highest dice score (0.79), where weights for B‐mode and SE‐mode modalities were selected automatically. The breast US images were cropped using the proposed segmentation model, where the lesion occupied most of the image. The proposed classification method with the cropped data set showed a sensitivity of 100% and specificity of 94.28%.

## 3 METHODOLOGY

**FIGURE 1:**
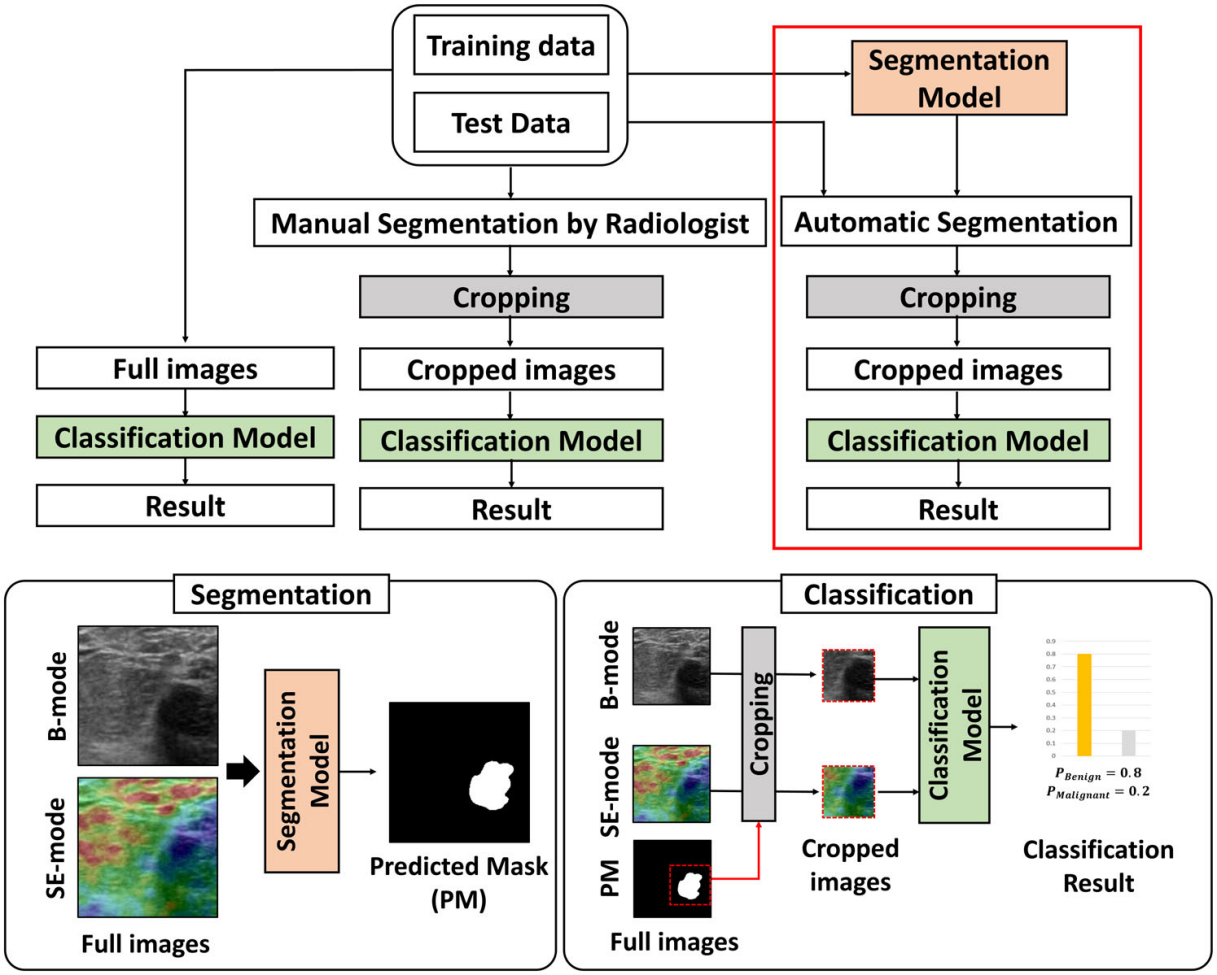
The generic flowchart of the proposed method

The generic flowchart of the proposed method is shown in Figure 1.

### 3.1 Data set

We have used the clinical breast US data set to validate the proposed framework. It was obtained from 73 patients at Western Reserve Health Education, Youngstown, OH, USA. The study was retrospectively approved by Institutional Review Board (IRB) and Health Insurance Portability and Accountability Act (HIPAA). The images were acquired between August 2016 and February 2017 using Hitachi's HI‐VISION Ascendus system with a linear‐type, 18–5 MHz EUP‐L75 probe. A total of 212 sets of bi‐modal US images were collected. Each set contains two modalities (B‐mode and SE‐mode) obtained from the same patient. Biopsies were performed for all patients, and the biopsy results were treated as the ground truth. There are 37 benign cases and 36 malignant cases. SE‐mode image is generally recorded accompanied by a B‐mode image for facilitating the visualization of the underlying structure. The details on obtaining SE‐mode images can be found in Refs. 6, 24. The data pre‐processing steps are shown in Figure S1, and the distribution of the data set is shown in Table S1. Patient details are in Table S2 (benign) and Table S3 (malignant). We arbitrarily divided the data set at the patient level into 137, 34, and 41 sets for training, validation, and testing, respectively for segmentation and classification.

All images are resized to a fixed image size of 336 × 336 pixels creating a reference data set without using any segmentation mask. We refer to this image as the “full image” in the paper. Segmentation is performed either manually by a radiologist or automatically by the proposed method. After segmentation, cropping is done either based on manual segmentation or based on the proposed segmentation model. For cropping, we have used a margin of 50 pixels. The full images are cropped either to a mask shape or rectangular shape. An example image of the full image and cropped images based on different cropping strategies is shown in Figure S2.

### 3.2 Segmentation mask

**FIGURE 2:**
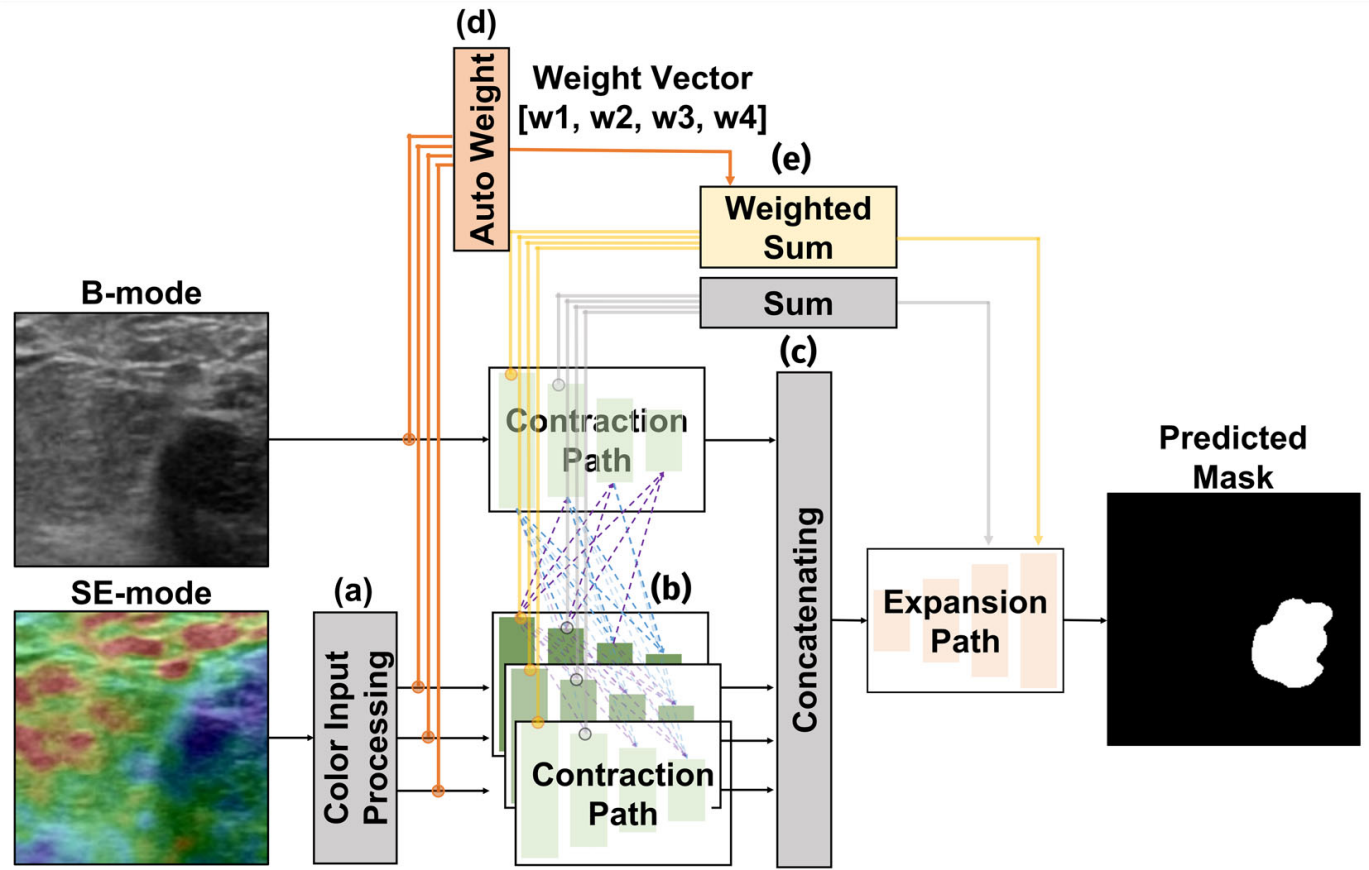
Diagram of the W‐MM‐U‐Net network. (a) The color input processing layer is included for converting color information into multiple modal information. (b) In the contraction path, layers are densely connected with another modal path layer. (c) The expansion path input is concatenating the result of contracted features. The concatenating layer can combine multiple important features from multiple modals with this layer. Then, the image is expanded to the original image size in the expansion path. (d) Unlike the original U‐Net, the weighted sum result obtained by passing the input image through the classification network is used as skip‐connection. (e) The weighted skip‐connection method is used only in the uppermost layer and the original skip‐connection method was adopted for other layers.

The proposed W‐MM‐U‐Net network for segmentation is demonstrated in Figure 2. It uses U‐Net, the most popular CNN model for medical image segmentation, as its basic structure. Like U‐Net, the proposed model consists of contraction and expansion paths. We adopted the contraction path form of IVD‐Net^25^ to use multiple inputs. The color input processing layer is added to the initial input processing part. We also added a new weighted skip‐connection method for weighting important modals.

#### 3.2.1 The color input processing layer

The two US modals (B‐mode and SE‐mode), used as network inputs, have different image information. The B‐mode US images provide the high‐resolution anatomical structure of the breast lesion whereas the SE‐mode US images display tissue stiffness and hard lesion, representing the soft lesions with different colors.^26^ However, the segmentation method developed so far does not use B‐mode and SE‐mode US image information together.^27^ The multimodal U‐net (MM‐U‐Net) model employed both B‐mode and SE‐mode images as inputs for accurate lesion segmentation.

The color input processing layer checks whether the input is a color image, and if it is, then the color information is converted into multiple modal inputs. When the SE‐mode image is used as a multimodal input, the network can use color information to indicate the stiffness of the tissue as much as possible.

#### 3.2.2 Auto‐weight stage and multimodal contraction path

The MM‐U‐Net model does not assign separate weights to each input modality.^25^ So, we can manually assign weight to each modality. However, it is not accurate. The final segmentation result can be improved by assigning the appropriate weight to each modality. So, in the Auto‐weight stage, we automatically searched weight to reflect the importance of each modal before passing the input data through the MM‐U‐Net. Here, a classification network (ResNet^28^) is employed to find the most appropriate weights for each input. Input data with four channels were passed through the ResNet model, and the weight vector with four values was obtained. The detailed method of obtaining weights will be described in the next section.

The proposed model utilized a multimodal contraction path with multiple modal layer routes. Four contraction paths exist for each modal: one for B‐mode input and three for SE‐mode input. Each modal's contraction path gradually contracts the preceding layer's feature, similar to a U‐Net. However, unlike U‐Net, the multimodal contraction path is connected by a hyper‐dense connection. A dense connection is closely connected to all layers, not just the layer in front of it.^29^ This dense connection method showed better results than the general connection method since it could use all the information about the preceding layers and a regularizing effect. Due to these advantages, a dense connection is used in many segmentation challenges.^30^, ^31^ However, the dense connections method still has limitations because it cannot connect between multimodal contraction paths. Hyper‐dense connection is used in many segmentation challenges since it is good to learn complex relationships between several modalities.^32^, ^33^

#### 3.2.3 Expansion path with weighted skip‐connection

The expansion path of this model is similar to the U‐Net as it expands information while expanding the contracted image back to the original image size. The acquired image is expanded using skip‐connection^28^ to supplement the spatial information loss. However, unlike U‐Net, MM‐U‐Net has multiple contraction paths, as it has multiple skip connections. MM‐U‐Net combined skip connections from several contraction paths to convey information to expansion paths.^25^ This skip‐connection allows combining fine location information of the shallow layer with the global semantic details of the deep layer. The common way to combine multiple skip connections is to average information about all the contraction paths. However, averaging multiple skip connections method does not consider the appropriate weight of each modal path when transmitting the information of the contracting path to the extraction path. Thus, when combining with the first skip‐connection, the most appropriate weight found in the previous auto‐weight step is applied to the information in each contraction path and then combined. Due to the dense connection of the contraction paths, the weight was reflected only in the first skip‐connection, the low‐level feature, before the information for each contraction path was mixed.

### 3.3 Classification model

#### 3.3.1 Model description

**FIGURE 3:**
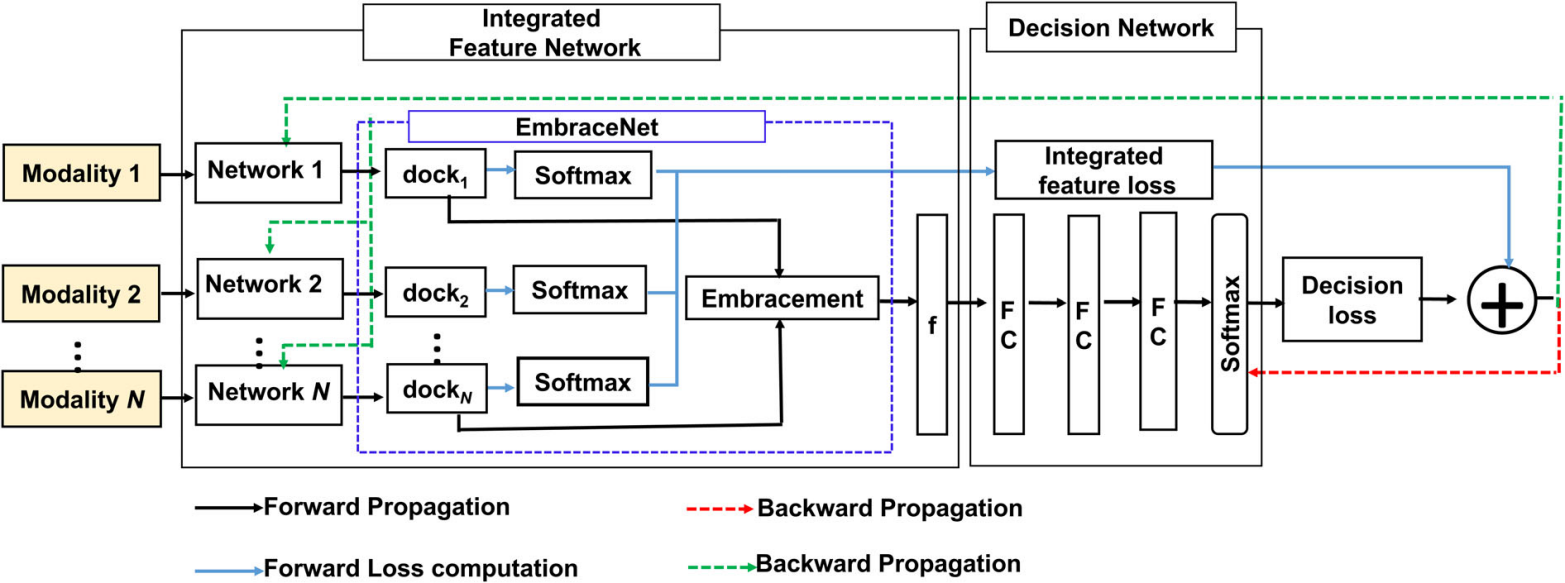
The outline of the proposed multimodal fusion framework. It is composed of the integrated feature network (IFN) and decision network (DN). The IFN comprises *N* network models and each model provides the integrated feature loss. Features of each network are ensembled to integrated features using EmbraceNet model to serve as input of the decision network (DN) for the breast US classification. All networks are optimized by the integrated feature loss and decision loss.

The schematic structure of the proposed MFF is shown in Figure 3. It comprises two sub‐networks: an integrated feature network (IFN) and a decision network (DN). The IFN comprises *N* network models with EmbraceNet^22^ models and softmax layers. It generates combined feature loss from network models for feature learning. The EmbraceNet model comprises docking and embracement layers. The output vectors of independent networks serve as inputs to the EmbraceNet model. The network model can be any network structure, for example, handcrafted feature vectors, multilayer perception, or CNN models. EmbraceNet transforms each vector to a dockable vector as the output vector size of the different network models can differ. In the embracement layer, the vectors obtained from docking layers are combined into a single vector. This single vector is known as the “embraced” vector. This embraced vector is served as the input of the DN. The DN comprises three FC (fully connected) layers and a softmax layer. Unlike recent deep ensemble methods, where the deep ensemble feature models and classifiers are trained before fine‐tuning the ensemble models, here, IFN and DN update simultaneously using integrated feature loss (*l*_*i*_) and decision loss (*l*_*d*_). The combined loss is backpropagated to the MFF in each epoch. Each network also has its classification (softmax) layer to calculate integrated feature loss to update both IFN and DN. The total loss function is defined as:

(1) $$l\left(\theta e,\theta d\right)=\mathrm{li}\left(\theta i\right)+\mathrm{\lambda ld}\left(\theta d\right),$$

where θi and θd are parameters for IFN and DN, respectively, and λ is the weight value.

Here cross‐entropy is utilized to compute the loss function of all networks in IFN. The integrated feature loss is calculated from all network models; thus, minimizing combined feature loss indicates reducing losses from all models. However, using only integrated feature loss is not enough because the classification results by each network may be incompatible due to each model having its softmax layer. This problem is addressed by taking the decision loss into account. The embraced vectors from all networks served as the input of the DN. The decision loss enforces the predicted labels to be consistent with true labels.

The probability threshold for both benign and malignant cases was set at 0.48. Therefore, any probability larger than 0.48 will classify the output to be classed as malignant, and any probability less than 0.48 will classify the output to be classified as benign. Threshold values were computed using the ROC curve and the optimal value was determined using Youden index.

#### 3.3.2 Implementation of model

##### Input modalities

The proposed MFF uses cropped B‐mode and SE‐mode US images as the input, and the classification result differentiating benign from malignant tumors is the output.

##### Transfer learning and pre‐trained models

In this paper, we employed CNN model as the network model. The CNN consists of input, output, and multiple hidden layers. The CNN‐based DL system takes input images with learnable weights and biases to differentiate one image from others. It is widely used in medical image classification tasks because of its excellent feature extraction capabilities. The accuracy of the CNN model depends upon extensive training data and layer design. For medical imaging, TL can be used with CNN models, where the models are pre‐trained using a large natural image data set (e.g., ImageNet). Although there is a large difference between the natural and medical image domains, low‐level features like edges, junctions, and corners learned from natural images can be transferred to the medical domain.

This paper employs the pre‐trained Visual Geometry Group‐16 (VGG‐16) model. This model was created by the Oxford Visual Geometry Group. It comprises 13 convolutional layers, five pooling layers, and three FC layers. Convolutional layers extract features, and 4096‐dimensional outputs of the FC layer describe features for classification.

It is worth noting that the two modalities share the same network to decrease over‐parameterization and the same consistency with other models.

#### 3.3.3 Multimodal Integration

##### Early integration^34^

**FIGURE 4:**
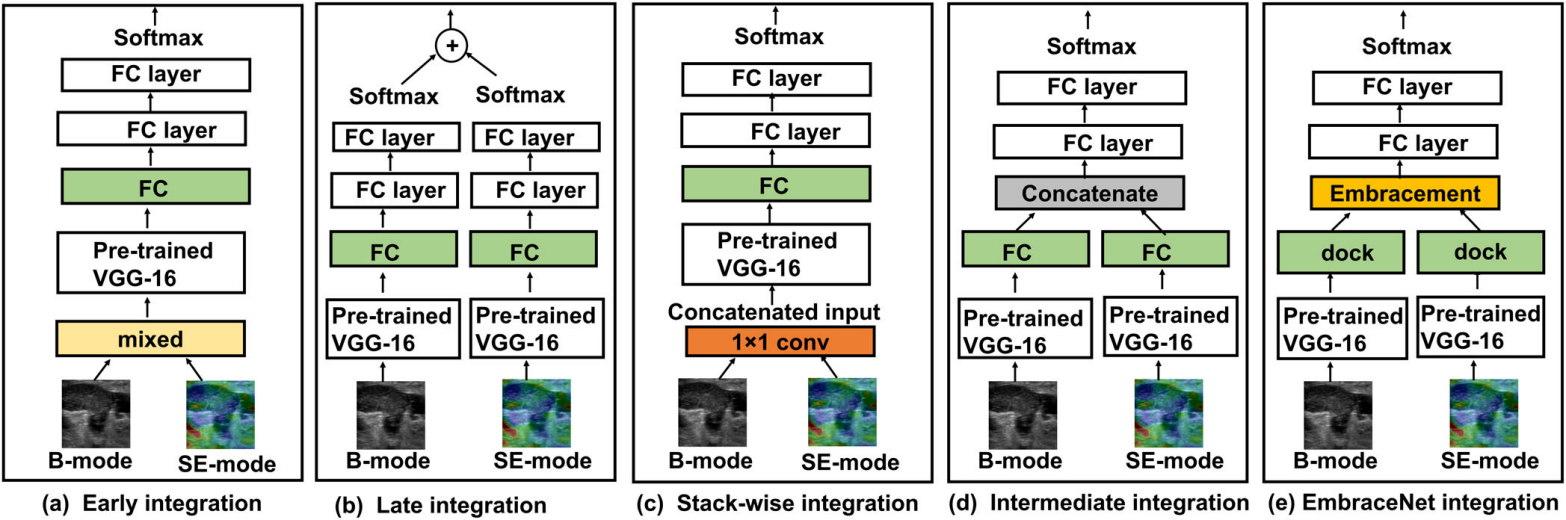
Structures of the multimodal network models employed on the breast US data sets

The early integration model is the most intuitive technique for multimodal evaluation. The structure of the early integration model is shown in Figure 4a. Here, B‐mode and SE‐mode US images are mixed into one data set for training. As the images from different modalities have different principal, the network learns complementary features from two different modalities. The input for the pre‐trained VGG‐16 model is the mixture of two modalities images. The classification result is then obtained from DN. US images from different modalities have different feature distributions, so, directly mixing two different modalities images may result in unobtrusive feature representations.

##### Late integration^35^

The late integration method fuses the decisions of models. Unlike the early integration method, separate classifiers are trained independently using images of different modalities in the late integration method. The final decision is taken by combining the outputs of the classifier. There are several ways to make the decision. As shown in Figure 4b, two independent pre‐trained VGG‐16 models are trained separately using B‐mode and SE‐mode US images for feature extraction. The final decision is determined based on the probabilities for the classifiers or soft voting.^36^

##### Stack‐wise early integration^6^

For the same original ultrasound image, its B‐mode and SE‐mode parts should be considered as different images. Therefore, two separate data modalities are combined into the model since their feature information is complementary. As shown in Figure 4c B‐mode (336 × 336 × 3 tensor) and SE‐mode (336 × 336 × 3 tensor) US images are fused into 336 × 336 × 6 tensor. Then, a 1 × 1 convolution is employed to reduce channel dimension and convert 336 × 336 × 6 tensor to 336 × 336 × 3 tensor, which is the input of the pre‐trained VGG‐16 model.

##### Intermediate integration

Here, learned features for various modalities are concatenated in the middle of the DL model. These networks handle not only multiple modalities in a single model but also consider mid‐level information among modalities. Here, B‐mode and SE‐mode US images are sampled independently and are inputted into two independent pre‐trained VGG‐16 models. The features extracted from these two models are concatenated. Thus, the output feature dimension of this model is twice that of a single model. The classification result is obtained from the DN based on the deep ensemble feature. As shown in Figure 4d, in intermediate integration, the part before the ensemble is similar to the lower part of late integration, and the part after the ensemble is identical to the upper part of early integration.

##### EmbraceNet integration^22^

The overall structure of EmbraceNet is similar to intermediate integration. The difference is in how features from pre‐trained models are used. The intermediate integration model typically concatenates the output of pre‐trained VGG‐16 models, each having 4096 features, and passes it to the DN layer. The EmbraceNet‐based model, on the other hand, passes the outputs of pre‐trained models to the EmbraceNet structure and then the DN layer, as shown in Figure 4e. The docking layer produces two output vectors of length 4096, and the embracement layer merges them into one.

### 3.4 Parameter setting and evaluation metrics

The optimal hyperparameters for single‐ and multimodal models were determined using the grid search. Various learning rates (0.01, 0.001, 0.0001), batch sizes (4, 8, 16, 32), and loss functions (binary cross entropy and dice loss) were used as hyperparameter to determine performance. The optimal hyperparameter values are shown in the third column of Table S4. This combination (learning rate/batch size/loss: 1e−4/32/dice) of hyperparameters performed near optimally for single and multimodalities. Thus, to enable fair comparison of results, we selected this set of hyperparameters to use for all models. The proposed segmentation method employed dice loss as a loss function (Equation 1),^37^ Adam optimizer with b1 = 0.9 and b2 = 0.999^38^ with an initial learning rate of 0.0001 and mini‐batch size of 32. We used fivefold cross‐validation to solve the overfitting problem. We obtained the highest score for selecting redundant results based on 2.5 thresholds from the fivefold cross‐validation results as the final result.^39^ The momentum and weight decay were set to 0.8 and 0.0001, respectively. We choose stochastic gradient descent (SGD) as the optimization solver. After both losses were calculated, the objective function in Equation (1) was optimized using SGD. The network was updated using back propagations (red and green dash lines), as demonstrated in Figure 3.

The performance of the proposed segmentation method was evaluated using four evaluation metrics: precision, recall, intersection over union (IOU), and dice coefficient. The IOU is obtained by the union of the predicted segmentation map and the ground truth. The dice coefficient is obtained by doubling the intersection of the predicted segmentation map and ground truth and dividing it by the sum of the two regions. We have used fivefold validation union results that determine and evaluate the region where more than half of the fivefold validation results overlap as the final region. The performance of the proposed classification method is evaluated based on accuracy, precision, specificity, sensitivity, and F1 score. Specificity and sensitivity predict benign and malignant lesions, respectively. Improving sensitivity is very important since it determines the detectability of malignant lesions.

## 4 RESULTS AND DISCUSSION

### 4.1 Segmentation

More than half of the fivefold validation results were named fivefold validation union results and used as the final result of cross‐validation.

#### 4.1.1 Quantitative comparison

An experiment was conducted to compare and analyze segmentation results based on the number and type of input modalities and the result is shown in Table 1. The results in the first and second rows show the performance of the segmentation method (U‐Net) using the B‐mode image and SE‐mode images, respectively. On the other hand, in rows 3–5, bi‐modal (both B‐mode and SE‐mode) images were used for input. The result in row 3 shows the performances of the segmentation method (MM‐U‐Net), where the same weight was given to both input modalities. The results in rows 4–5 show the performance of the proposed segmentation method (W‐MM‐U‐Net), where the optimum weight of input modalities was assigned manually and automatically based on their importance. Overall, the segmentation results of bi‐modal models are better than single‐modal models. This MM‐U‐Net model showed better results (0.77 dice score) than the U‐Net model using only B‐mode image (0.72 dice score) or the U‐Net model using only SE‐mode image (0.76 dice score). It shows that it is better to use two modals than only B‐mode or only SE‐mode input images because these two modalities complement each other's information and show better results. Functional information in the SE‐mode provides information about areas of interest to perform segmentation, while structural information in the B‐mode has helped to find accurate Lesion boundary information. Further, performance is improved by applying appropriate weights (0.77 for manual W‐MM‐U‐Net and 0.79 for automatic W‐MM‐U‐Net) to different input modalities based on their importance. So, we employed automatically selected weight to our MM‐U‐Net network using the weighted skip‐connection method. This W‐MM‐U‐Net (automatic) method is expected to find optimal weights automatically, even for the new data set.

**TABLE 1:** Cross‐validated segmentation results using different input modality and network

Input Modality	Network	Dice‐coefficient (%)	IOU (%)	Precision (%)	Recall (%)
B‐mode	U‐Net	67.20 ± 2.90 (71.7)	53.42 ± 3.46 (58.5)	61.86 ± 5.77 (67.8)	83.26 ± 2.47 (84.2)
SE‐mode	U‐Net	67.86 ± 8.46 (76.3)	54.42 ± 9.24 (63.9)	62.22 ± 13.43 (72.3)	86.26 ± 6.41 (86.5)
B‐mode + SE‐mode	MM‐U‐Net	73.44 ± 1.54 (77.3)	60.96 ± 1.62 (65.5)	75.04 ± 6.02 (79.3)	80.44 ± 5.70 (80.6)
B‐mode + SE‐mode	W‐MM‐U‐Net (manual)	74.96 ± 1.10 (77.8)	62.84 ± 1.60 (66.2)	77.26 ± 5.78 (81.2)	80.44 ± 7.13 (79.8)
**B‐mode + SE‐mode**	**W‐MM‐U‐Net (automatic)**	**74.68 ± 1.04 (78.8)**	**62.50 ± 1.19 (67.7)**	**74.74 ± 3.18 (80.3)**	**84.42 ± 1.70 (84.6)**

*Note*: All metrics are reported in mean ± SD format, while the fivefold validation union results are in parentheses. The proposed method and its performance values are highlighted in bold.

Segmentation results for different input modalities and networks with optimal hyperparameters are shown in Table S4. It is worth noting that the optimal multimodal model outperformed the optimal single‐modal model on the test set for all of the optimal hyperparameters.

#### 4.1.2 Qualitative comparison

**FIGURE 5:**
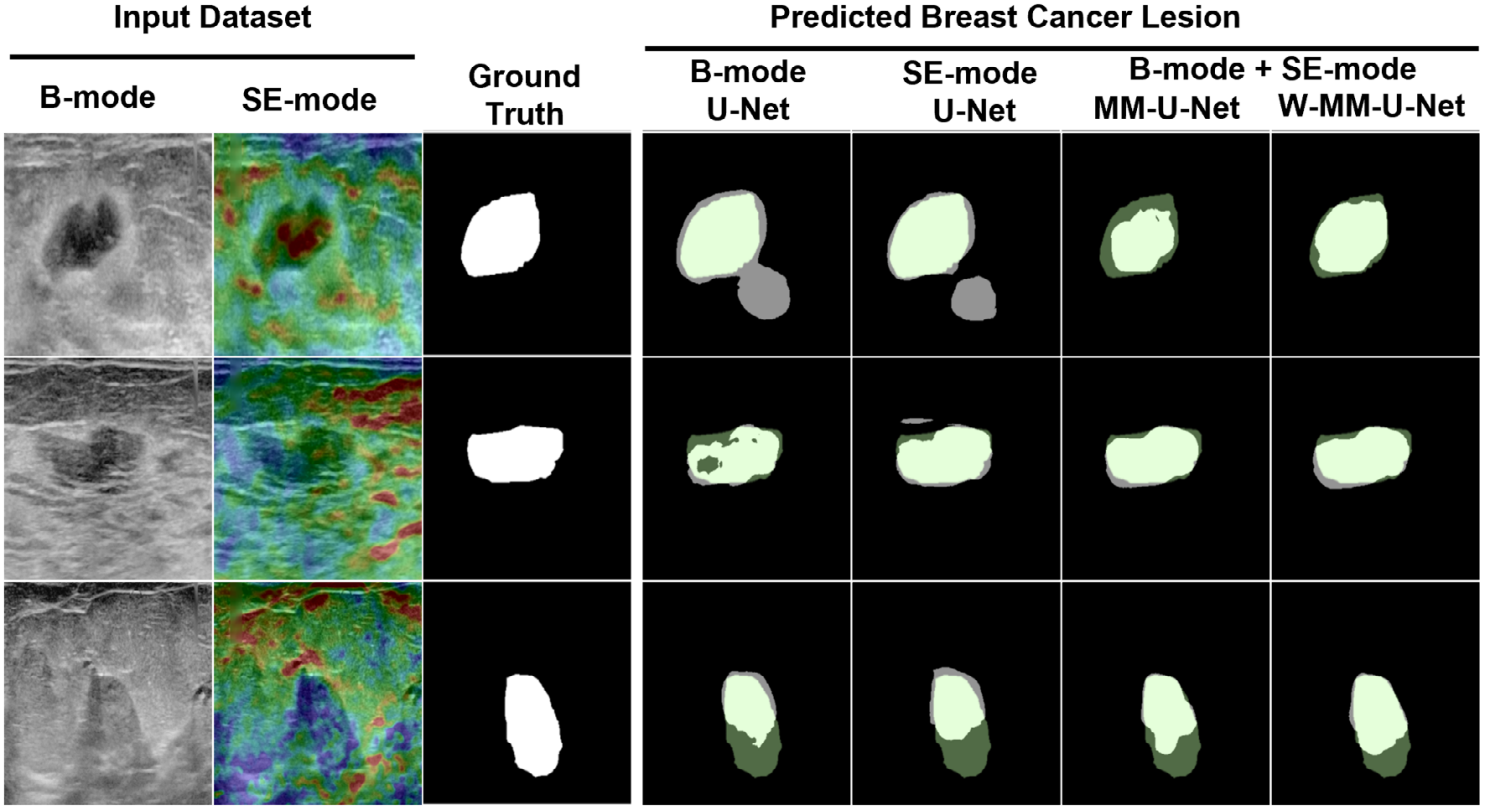
Qualitative comparison of different segmentation networks by visualization. From left to right: B‐mode/U‐Net, SE‐mode/U‐Net, B‐mode and SE‐mode/MM‐U‐Net, B‐mode and SE‐mode/W‐MM‐U‐Net. In the predicted mask, the green part is the ground‐truth part, and the gray part is the predicted part. The bright green part is precisely predicted part.

The qualitative comparison of several segmentation models is shown in Figure 5. The first, second, and third columns show the examples of B‐mode, SE‐mode, and their corresponding ground truth (by radiologist) images. The predicted mask using single‐modal and bi‐modal models are shown in columns 3–4 and columns 5–6, respectively. In many cases, the non‐lesion part was predicted using single‐modal images. However, the prediction was more similar to ground truth when bi‐modal inputs were used. The best segmentation mask is shown in the case of the proposed W‐MM‐U‐Net.

We have used the predicted segmentation mask from the proposed W‐MM‐U‐Net model to automatically crop the lesion part from the “full image” as the performance of the proposed model is improved than the other models.

### 4.2 Classification

The cross‐validated classification performance is shown patient‐wise. Majority voting is used to predict the particular class of patients. However, in case of a tie, priority is given to the malignant class.^6^

#### 4.2.1 Single modal versus bi‐modal

**FIGURE 6:**
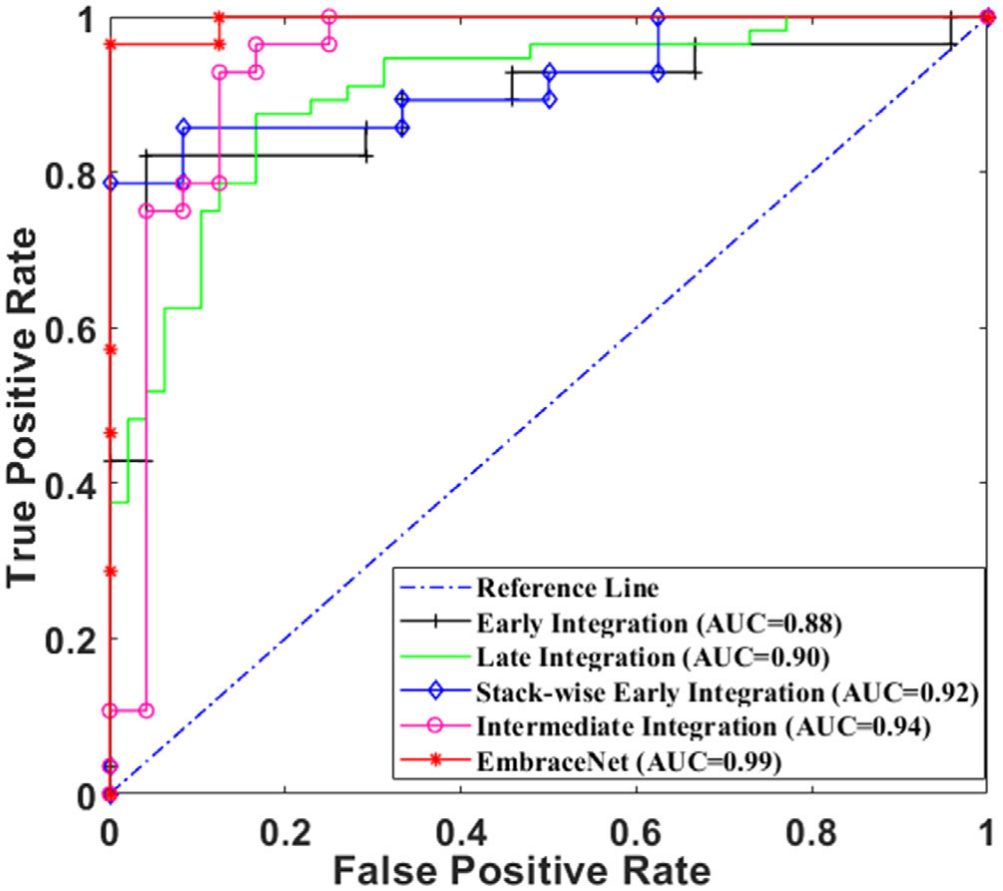
Receiver operating characteristic (ROC) curves for classification of breast US image using multimodal classification methods

Table 2 shows the patient‐wise cross‐validated results of comparative experiments. The results in the first row show the performance of the proposed classification method (MFF) using full B‐mode and SE‐mode images (without using any segmentation masks), where modality integration was done using the EmbranceNet model. The results in the second row show the performance of the MFF using cropped B‐mode and SE‐mode images where the manual segmentation mask (by a radiologist) was used for cropping. On the other hand, in rows 5–9, the proposed segmentation mask (W‐MM‐U‐Net) is used for cropped images. The results in rows 3–4 and rows 5–9 show the classification performances using single modality (B‐mode or SE‐mode) images and bi‐modal images, respectively. Overall, bi‐modal models score better than single‐modal models. It validates that complementary information is available among B‐mode and SE‐mode images and is beneficial to incorporate multiple available information to improve performance. Note that, the performance of the model trained using SE images is superior to the model trained using B‐mode images. This indicates that tissue stiffness is an important factor to identify malignant lesions. The performance of the early integration method is inferior to various integration methods as directly mixing two different modalities images (B‐mode and SE‐mode) may result in unassuming feature representations. Overall, the MFF using the EmbraceNet integration model outperforms the early, late, stack‐wise, and intermediate integration. The EmbraceNet‐based MFF model achieved the classification accuracy of 96.92 ± 3.77% using cropped B‐mode and SE‐mode images. The W‐MM‐U‐Net model was used for the segmentation mask. For the 13 test cases (7 benign and 6 malignant), the proposed method predicted 7 benign patients as benign three times out of five trials and 6 malignant patients as malignant five out of five trials. So, the proposed method predicted all 13 test patients correctly 3 times (100%) and 1 patient incorrectly 2 times (92.31%). We employed the paired *t*‐test^40^ to specify the statistical significance of our results across different evaluation metrics, as shown in Table 2. The performance of the proposed EmbraceNet‐based MFF model is statistically validated against other single‐modality models and state‐of‐the‐art multimodal models by considering the null hypothesis that the performance of the proposed model was equivalent to other models. The *p*‐values are provided for a 95% confidence interval and the significance is denoted by two signs: +, which indicates that the performance of the proposed model is significantly better (i.e., *p* ≤ 0.05, rejecting the null hypothesis), and ≈, which indicates that the performance of the proposed and the other models is equivalent (i.e., *p* > 0.05, which cannot reject the null hypothesis). The area under the curve (AUC) values and the ROC curves of the various bi‐modal models are shown in Figure 6. The proposed model accomplished the best performance with an AUC of 0.98 among all the existing models.

**TABLE 2:** Cross‐validated classification results of different methods

Image (modality)	Segmentation	Network (integration)	Accuracy (%)	Precision (%)	Specificity (%)	Sensitivity (%)	F1‐score (%)
Full image (B‐mode + SE‐mode	‐	MFF (EmbraceNet)	81.54 ± 3.77 (+)	80.28 ± 4.61 (+)	82.85 ± 5.71 (+)	79.99 ± 6.66 (+)	79.93 ± 4.37 (+)
Cropped image (B‐mode + SE‐mode	Manual segmentation	MFF (EmbraceNet)	92.31 ± 0.00 (+)	**97.14 ± 5.71 (≈)**	**97.14 ± 5.71 (≈)**	86.66 ± 6.67 (+)	91.19 ± 0.56 (+)
Cropped image (B‐mode)	W‐MM‐U‐Net	VGG‐16 (single)	72.31 ± 6.15 (+)	70.00 ± 6.66 (+)	74.29 ± 5.71 (+)	70.00 ± 6.66 (+)	70.00 ± 6.66 (+)
Cropped image (SE‐mode)	W‐MM‐U‐Net	VGG‐16 (single)	81.54 ± 3.77 (+)	78.57 ± 5.82 (+)	79.99 ± 6.99 (+)	83.33 ± 0.00 (+)	80.77 ± 3.14 (+)
Cropped image (B‐mode + SE‐mode)	W‐MM‐U‐Net	MFF (early)	83.08 ± 3.08 (+)	80.95 ± 4.76 (+)	82.85 ± 5.71 (+)	83.33 ± 0.00 (+)	82.05 ± 2.56 (+)
Cropped image (B‐mode + SE‐mode)	W‐MM‐U‐Net	MFF (late)	86.16 ± 3.08 (+)	83.81 ± 0.95 (+)	85.71 ± 0.00 (+)	86.67 ± 6.67 (+)	85.13 ± 3.59 (+)
Cropped image (B‐mode + SE‐mode)	W‐MM‐U‐Net	MFF (stack‐wise)	90.77 ± 3.07 (+)	90.95 ± 7.44 (≈)	91.43 ± 7.00 (≈)	89.99 ± 8.16 (+)	86.92 ± 4.39 (+)
Cropped image (B‐mode + SE‐mode)	W‐MM‐U‐Net	MFF (intermediate)	92.31 ± 0.00 (+)	94.28 ± 7.00 (≈)	94.28 ± 7.00 (≈)	89.99 ± 8.16 (+)	91.47 ± 0.69 (+)
**Cropped image (B‐mode + SE‐mode)**	**W‐MM‐U‐Net**	**MFF (EmbraceNet)**	**96.92 ± 3.77**	94.28 ± 7.00	94.28 ± 7.00	**100 ± 0.00**	**96.92 ± 3.77**

*Note*: All metrics are reported in mean ± SD format, while the best results are highlighted in bold. +, statistically significant; ≈, statistically not significant.

Abbreviation: MFF, multimodal fusion framework.

#### 4.2.2 Comparison with existing methods

We have compared the proposed method with other existing state‐of‐the‐art techniques,^6^, ^16^, ^41^, ^42^, ^43^, ^44^ and the results are shown in Table 3. The data set used for the existing methods is the same full breast US images as used in this study. The two‐tailed paired *t*‐test^40^ has been employed to statistically validate all of the results for test cases, under the null hypothesis that the performance of the proposed approach is equivalent to that of other existing methods. The *p*‐values are provided for a 95% confidence interval and the significance is denoted by two signs: +, which indicates that the performance of the proposed method is significantly better (i.e., *p* ≤ 0.05, rejecting the null hypothesis), and ≈, which indicates that the performance of the proposed and the existing methods is equivalent (i.e., *p* > 0.05, which cannot reject the null hypothesis). It could be noted that the *p*‐values on the performance measures provide evidence for the statistical significance of the proposed EmbraceNet‐based MFF method. This is mainly because the proposed method utilized features from both B‐mode and SE‐mode images and reflected cross‐modal correlations accurately.

**TABLE 3:** Classification performance of our proposed method and existing methods

Existing methods	Accuracy (%)	*p*	Precision (%)	*p*	Specificity (%)	*p*	Sensitivity (%)	*p*
Yap et al.^16^	72.31 ± 3.76	0.00001	68.57 ± 2.33	0.00006	71.83 ± 0.49	0.00010	73.33 ± 8.16	0.00009
Fujjoka et al.^40^	72.31 ± 3.76	0.00001	73.69 ± 5.99	0.00104	79.99 ± 6.99	0.01015	63.34 ± 6.67	0.00001
Zhang et al.^41^	75.38 ± 5.75	0.00012	75.33 ± 7.17	0.00269	79.99 ± 6.99	0.01015	70.00 ± 6.66	0.00001
Kumar et al.^42^	76.92 ± 6.88	0.00047	76.00 ± 7.71	0.00398	79.99 ± 6.99	0.01015	73.33 ± 8.16	0.00001
Wang et al.^43^, ^a^	87.69 ± 3.76	0.00426	87.14 ± 6.49	*0.08649*	8856 ± 5.71	*0.12075*	86.66 ± 6.66	0.00197
Misra et al.^6^	90.77 ± 3.07	0.01763	90.95 ± 7.44	*0.26612*	91.43 ± 7.00	*0.28979*	89.99 ± 8.16	0.01998
**Proposed**	**96.92 ± 3.77**		**94.28 ± 7.00**		**94.28 ± 7.00**		**100 ± 0.00**	

*Note*: The bold emphasizes the proposed method and its performance values. Significant test results (*p*‐values) attained by proposed method against existing methods for all performance metrics. The values in italics indicate statistically not significant results.

^a^ We have used only B‐mode and SE‐mode images.

### 4.3 Limitation and future scope

In general, DL models achieve outstanding performances with a large number of training images. The main limitation of developing a supervised DL model for a medical imaging task is the lack of access to a sizable, labeled data set. Therefore, it is important to design solutions that work with fewer training samples to expand the usability of DL method. Data augmentation, pre‐trained model adaptation, and fine‐tuning have been applied in the proposed method to learn the high‐dimensional feature from S3 and achieved superlative performances. As and when more data becomes available, it is possible to augment our training model further to achieve further improvement in performance. But current performance, as it stands, establishes that even with small amount of data DL machine could add significant value to clinical practice.

Another drawback of this study is its retrospective design, and the single‐center character which leaves room for selection bias. For different centers or different sets of data set, our model could be used as a base model upon which performance can be evaluated and improvements to be made quickly. Performance of the proposed method should be evaluated prospectively before its implementation in clinical practice.

## 5 CONCLUSION

In this paper, we propose (a) a novel deep learning‐based weighted multimodal U‐Net (W‐MM‐U‐Net) model using B‐mode and SE‐mode US images to segment the cancer lesion and (b) a fusion network (MFA) that can process two types of US modalities for the differentiation of benign and malignant breast lesions. The W‐MM‐U‐Net is based on the IVD‐Net model where segmentation can be performed using multimodal input. Unlike other models, the proposed W‐MM‐U‐Net model automatically assigns appropriate weight to the input modalities based on their importance. The MFA is based on the EmbraceNet model, which combines multimodal information and deals with the correlation between two modalities. Compared to other fusion networks, the proposed MFA can simultaneously learn features from the embracement feature network and decision network for better classification performance. The proposed model reflects cross‐modal correlations and efficiently prevents overfitting because of its regularized learning process. The experimental results indicate that the performance of the proposed method is significantly improved than other single‐modality models and other state‐of‐the‐art multimodal algorithms. In the future, we will adopt this method for more modalities of US images (like SWE, color doppler). In addition, as the outline of this framework is common, we will expand this method to other medical assignments for clinical treatment.

## AUTHOR CONTRIBUTIONS

**Sampa Misra:** Conceptualization (equal); data curation (equal); formal analysis (equal); methodology (equal); software (equal); validation (equal); writing – original draft (equal); writing – review and editing (equal). **Chiho Yoon:** Conceptualization (equal); data curation (equal); methodology (equal); software (equal); writing – original draft (equal); writing – review and editing (equal). **Kwang‐Ju Kim:** Conceptualization (equal); formal analysis (equal); investigation (equal); supervision (equal); writing – review and editing (equal). **Ravi Managuli:** Data curation (lead); supervision (equal); writing – review and editing (equal). **Richard G. Barr:** Data curation (equal); supervision (equal); writing – review and editing (equal). **Jongduk Baek:** Supervision (equal); writing – review and editing (equal). **Chulhong Kim:** Funding acquisition (lead); investigation (equal); project administration (lead); supervision (lead); writing – review and editing (equal).

## CONFLICT OF INTEREST

The authors declare no competing financial or non‐financial interests.

### PEER REVIEW

The peer review history for this article is available at https://publons.com/publon/10.1002/btm2.10480.

## Supporting information


**Appendix S1.** Supporting Information.

## Data Availability

Data sets will be made available to the requested after due process when requested from the first author. Since its clinical data institution has to agree based on the request made.
