# Supplementary material for: Deep learning‐based multimodal fusion network for segmentation and classification of breast cancers using B‐mode and elastography ultrasound images
Source: Bioeng Transl Med. 2022 Dec 28;8(6):e10480. doi: 10.1002/btm2.10480 (PMC10658476; doi:10.1002/btm2.10480)
Supplement: Supplementary file 1 — Appendix S1. Supporting Information. [file BTM2-8-e10480-s001.docx]

Supplementary Information for:

**Deep Learning-based Multimodal Fusion Network for Segmentation and Classification of Breast Cancers using B-mode and Elastography Ultrasound Images**

Sampa Misra^1†^, Chiho Yoon^1†^, Kwang-Ju Kim^2†^, Ravi Managuli^3^, Richard G Barr^4^, Jongduk Baek^5^, and

Chulhong Kim^1*^

^1^Department of Electrical Engineering, Convergence IT Engineering, Mechanical Engineering, Medical Device Innovation Center, and Graduate School of Artificial Intelligence, Pohang University of Science and Technology, Pohang 37673, South Korea.

^2^Electronics and Telecommunications Research Institute, Daegu, South Korea.

^3^Department of Bioengineering, University of Washington, Seattle 98195, USA.

^4^Southwoods Imaging, 7623 Market Street, Youngstown, OH 44512, USA.

^5^School of Integrated Technology, Yonsei University, South Korea.

(*Corresponding author: *Chulhong Kim;*[chulhong@postech.edu](mailto:chulhong@postech.edu)).

(^†^Equal contribution)

**Table of contents**

**Supplementary Figures**

Supplementary Fig. 1. Example of B-mode and SE-mode images and dataset preprocessing steps2

Supplementary Fig. 2. Example of the full image and cropped images 3

**Supplementary Tables**

Supplementary Table 1. Patient-wise and image-wise class distributions 4

Supplementary Table 2. Details of benign patients.5

Supplementary Table 3. Details of malignant patients.6

Supplementary Table 4. Segmentation results using different input modalities and networks with optimal

hyperparameters7

Fig. 1. Example of B-mode and SE-mode images and dataset preprocessing steps. (a) Full screenshot image with SE-mode and B-mode US image. (b) SE-mode and B-mode cropped image with marks & color bars. (c) SE-mode and B-mode mark removed image. (d) Dataset preprocessing steps.

Fig. 2 shows example of the full image and cropped images based on different cropping strategies. Rect, Rectangular shape; mask, Mask shape.

Table I. Patient-wise and image-wise class distributions (train validation, and test sets). All images acquired from a specific patient belong exclusively to either test or validation; no images are allocated to both test and validation.

|  | Dataset | | | | | |
| --- | --- | --- | --- | --- | --- | --- |
|  | Total | | Benign | | Malignant | |
|  | Patients | Images | Patients | Images | Patients | Images |
| Total | 73 | 212 | 37 | 113 | 36 | 98 |
| Train | 48 | 137 | 24 | 76 | 24 | 60 |
| Validation | 12 | 34 | 6 | 19 | 6 | 15 |
| Test | 13 | 41 | 7 | 18 | 6 | 23 |

**Supplementary Table 2.** Details of benign patients.

| **PT #** | **Type** | **Strain Ratio** | **PT #** | **Type** | **Strain Ratio** | **PT #** | **Type** | **Strain Ratio** |
| --- | --- | --- | --- | --- | --- | --- | --- | --- |
| 201 | fat necrosis | 4.35 | 244 | fibroadenoma | 1.52 | 277 | fibrosis | 4.57 |
| 203 | fibroadenoma | 1.75 | 246 | fat necrosis | 5.84 | 279 | fibrosis | 0.75 |
| 207 | intraductal papilloma | 3.81 | 249 | fibrocystic | 5.19 | 280 | fibroadenoma | 3.8 |
| 208 | lipoma | 0.81 | 251 | fibroadenoma | 4.4 | 281 | fibroadenoma | 2.48 |
| 211 | fibroadenoma | 6.17 | 256 | complex cyst | 2.54 | 283 | fibrocystic | 1.23 |
| 215 | fat necrosis | 3.96 | 264 | fibroadenoma | 4.28 | 284 | fibrocystic | 3.33 |
| 224 | benign lymph node | 0.96 | 265 | fibroadenoma | 2.64 | 285 | fibroadenoma | 2.42 |
| 228 | fibroadenoma | 3.57 | 266 | fibrocystic | 4.98 | 286 | fibroadenoma | 10.22 |
| 230 | fibro change | 3.67 | 267 | fat necrosis | 9.66 | 287 | fibroadenoma | 2.09 |
| 233 | fibroadenoma | 1.29 | 269 | hyperplasia | 1.37 | 290 | benign lymph node | 3.32 |
| 234 | fibroadenoma | 1.7 | 272 | fibroadenoma | 2.06 | 291 | fibroadenoma | 2.49 |
| 237 | hyperplasia | 1.97 | 273 | hematoma | 0.38 |  |  |  |
| 243 | fibroadenoma | 1.63 | 276 | fibrocystic | 2.63 |  |  |  |

**Supplementary Table 3.** Details of malignant patients. [IDC, Invasive Ductal Carcinoma; Ca, Carcinoma; DCIS, Ductal Carcinoma in Situ; ILC, Invasive lobular carcinoma](https://www.hopkinsmedicine.org/health/conditions-and-diseases/breast-cancer/invasive-ductal-carcinoma-idc#:~:text=Invasive%20ductal%20carcinoma%2C%20also%20known,of%20all%20breast%20cancer%20diagnoses.)

| **PT #** | **Type** | **Strain Ratio** | **PT #** | **Type** | **Strain Ratio** | **PT #** | **Type** | **Strain Ratio** |
| --- | --- | --- | --- | --- | --- | --- | --- | --- |
| 202 | Invasive papillary  cancer | 1.36 | 219 | IDC and DCIS | 5.87 | 254 | IDC and DCIS | 7.57 |
| 204 | IDC | 2.15 | 220 | IDC and DCIS | 4.98 | 255 | ILC | 13.99 |
| 205 | IDC | 3.04 | 221 | IDC | 15.78 | 258 | IDC | 10.96 |
| 206 | IDC | 7.29 | 222 | IDC | 4.69 | 259 | IDC -triple - | 2.96 |
| 209 | IDC | 14.03 | 223 | IDC | 7.50 | 261 | signet cell ca | 6.32 |
| 210 | IDC | 6.07 | 225 | IDC | 4.11 | 268 | IDC | 12.61 |
| 212 | IDC | 5.95 | 227 | IDC | 3.45 | 270 | IDC | 2.59 |
| 213 | IDC | 7.77 | 235 | IDC | 7.62 | 271 | IDC | 1.79 |
| 214 | tubular Ca | 2.65 | 236 | IDC | 4.79 | 274 | IDC | 13.98 |
| 216 | IDC | 6.35 | 238 | IDC | 12.34 | 275 | IDC | 4.42 |
| 217 | IDC | 3.52 | 248 | IDC mucinous | 4.2 | 282 | papilloma with minimal IDC | 6.71 |
| 218 | IDC | 18.61 | 250 | IDC | 3.22 | 289 | IDC micropapillary | 6.82 |

**Supplementary Table 4.** Segmentation results using different input modalities and networks with optimal hyperparameters. lr, learning rate; bs, batch size; bce, binary cross entropy.

| **Input Modality** | **Network** | **Optimal values**  **(lr/bs/loss)** | **Dice-coefficient (%)** | **IOU**  **(%)** | **Precision**  **(%)** | **Recall**  **(%)** |
| --- | --- | --- | --- | --- | --- | --- |
| **B-mode** | **U-Net** | **1e-4 / 16 / dice** | **63.6** | **50.5** | **88.6** | **57.4** |
| **SE-mode** | **U-Net** | **1e-3/ 8 / dice** | **58.1** | **45.3** | **90.7** | **48.5** |
| **B-mode + SE-mode** | **MM-U-Net** | **1e-4 / 32 / dice** | **68.1** | **55.0** | **85.4** | **65.2** |
| **B-mode + SE-mode** | **W-MM-U-Net (manual)** | **1e-4 / 32 / dice** | **66.5** | **53.5** | **86.2** | **62.8** |
| **B-mode + SE-mode** | **W-MM-U-Net (automatic)** | **1e-4 / 8 / bce** | **69.1** | **57.3** | **82.3** | **67.9** |
